# Mal-Occlusion of the Teeth and Its Intimate Relations to Carious Conditions

**Published:** 1906-10-15

**Authors:** F. E. Williams


### MAL-OCCLUSION OF THE TEETH AND ITS INTIMATE RELATIONS TO CARIOUS CONDITIONS.

BY F. E. WILLIAMS, D.D.S.

Read before the Michigan Dental Association, July 11, 1906.

In examining the cases coming under immediate attention we are cognizant and more or less concerned that patients who are quite young, (from six years old even to adult age) are affected with diseased conditions of the mouth and while carious teeth are the rule and are to be expected, we find other conditions outside of that, common and arising from the same cause. We expect and are looking for tooth decay more in the child than the adult, when the tooth structure and all the tissues have attained to their best condition, or should have if not too much handicapped by disease, and are in a position to better combat disease, having preserved their strength. Surplus vitality having been stored up may be used when needed for repair of tissues, but not as growth.

We, in our position, are in duty bound to make conditions as we find then as normal as possible, for the strength of the adult organism is immediately dependent upon the perfection of its structure.

Now in the beginning where shall we start? We cannot wait to see what nature is going to do for us in the future, we can only see nature is doing her best under prevailing conditions, trying to get away from the irritation by absorbtion and to overcome it by excessive building of new structure, new tissue. Then our beginning should be with the origin of the pathogenic tendency, for at that time we can do the most good; the embryonic structure is at its best and responds more readily to treatment, it being in the growing stage the length of treatment is necessarily much shorter.

Mal-occlusion is a prime mover in the carious conditions of the mouth. First the teeth being out of position, that is, not being in the proper occlusion. They are protruding, bunched, (which is classified as retruding) and turned, and the fact of their being in that mal-position is sufficient to bring the contigious membrane into an abnormal condition.

The dental ligiments are not in proper condition or position. They are stretched around the teeth in different positions, causing an obstruction to the circulation by a continual tension on the blood vessels also hindering the elimination of waste products, as well as the bringing of nourishment to these tissues. In this position the gums look thick, the circulation being sluggish, as is shown by the dark appearance of the gums. They may be swollen and standing away from the enamel of the teeth, covering it partly, giving an unsightly appearance, impossible to keep clean as they should be and a case where no amount of treatment, prophylactic or otherwise could materially remedy the condition.

This condition is found in all cases except where the teeth are regularly protruding and rather than there being too much gum tissue, there is not enough, for it is in a stretched condition and may be seen in any individual disarrangement of the teeth. The muscular fibers and all the tissues, are less than their normal amount in bulk and respond more readily to irritants while in this condition. There is no retention of food particles as there are no retaining spaces, but the gums recede and there is decided retention owing to the shoulder of process and gums resulting. In other conditions the gums are loose and a great factor in the retention of food particles around the teeth. These pockets make it impossible to keep the teeth clean and greatly enhance the chances of decay, and later the advance agent of hypertrophy and deposits on the teeth, resulting in recession of the gums,inflammation of the gums and peridental membrane and pyorrhea with ultimate loss of teeth.

Children do not take as good a care of their teeth as they do later, but many try their best to keep their mouths in a cleanly condition and yet fail, because it is more difficult on account of mouth breathing and mal-occlusion. It is noticable that the portion of the mouth where the teeth are regular will be in a good condition and with much less care. In regular dentures the festoons of the gums hugg the teeth closely not allowing the retention of food except at the approximal space, but which may be cleaned easily, because the surface of the teeth are in a high state of polish and all the surfaces sloping gradually away.

Where the teeth are irregular the surfaces are more or less roughened, caused from contact with food retained and its action, also the retained constituents of the saliva. The food remaining against the teeth continually, and from this contact and chemical action from fermentation the enamel is acted upon and becomes roughened and after a time breaks down. The gums and process recede, diseased conditions setting in which results sooner or later in the loss of the affected teeth, sacrificed by what amounts to carelessness and neglect.

Perhaps the most frequent place we notice the foregoing condition is in the lower incisor teeth. They are because of their location and position peculiarly liable to these conditions also because of the shape and size of their roots and their frail supporting tissues, we find them peculiarly liable to every trouble except decay. We also notice this condition of the gums in many other teeth, according to their location and degree of mal-occlusion. The cuspid teeth are many times protruding or too prominent and in this condition respond readily to irritation, the gums and process receding according to the degree of irritation, showing a large portion of the tooth, and becoming very sensitive at times to thermal changes and to the touch. On the other hand the tooth being in lingual occlusion or the teeth approximate to it being in buccal or libial occlusion, food is retained and we are liable to lose the tooth entirely, by pyorrhea or death of the peridental membrane, if the case is not corrected. Furthermore the food is the more readily retained after absorption has begun and proceeds more rapidly. The enamel having a hard smooth surface is not attacked so easily, but the dentine on exposure presents a roughened surface which aids greatly in retaining any thing coming in contact with it; it will be exposed to atmospheric conditions instead of being covered by the process and peridental membrane and gums as in a normal condition. Contact with food, and not having the smooth surface of the enamel to protect it, a good surface for the lodgment of food and attack of decay is provided and it is also very hard to clean. In these conditions special care must be taken for the gums are receding and the added irritation in extra cleaning, is sufficient for the further receding of the gum and absorption of process.

In the case of children, not only is food the more readily retained but we have actual loss of tissue while it is in a growing condition. We should have instead added strength and vitality the better to withstand attacks of disease in after life. Instead the strength is undermined in youth—leaving the tissue in a weakened condition and unable to contend with abnormal conditions, hence the sooner they succumb to disease and destruction.

We see these conditions in the little folks, who are too young for the disease of later life, at a time when the mouth should be in fine condition, even without the intelligent use of the tooth brush.

These tendencies do not end here, for the food held in contact with the teeth, gives the very best conditions for the operation of the germs of decay, and we have destruction of tooth structure as well as the gums and process. We have the germs of decay already in the mouth at an extremely early age, ready to attack the new structures at any and all times.

We may have absorption going on when the tissues should be growing and improving. More pockets formed for the retention of carious masses and added roughened surfaces along the teeth for the attack. Because of the mal-occlusion mastication also is faulty and this lessens the degree of perfection in mastication, thereby lowering the general vitality of the system, and causing abnormal conditions of the mucous membrane of the mouth and the alimentary tract. When the patient breathes through the mouth, the mucous membrane becomes dry and irritated by the constant passage of air through the mouth. The surfaces of the teeth becoming dry give an advantage to decay producing agencies instead of having the protection of the saliva as is normally the case.

We find the trouble greater with the anterior teeth, because the posterior teeth are more strongly supported than those anteriorly to them. We find mal-occlusion in the posterior teeth as a certainty and though we have to shift the occlusion many times, in order to get them into their proper position and they are harder to move, it is certain to meet with success and the outcome in such cases is satisfactory. But the posterior teeth are generally nearer to position than the anterior teeth, for in classes Two and Three as well as class One, the front teeth are forced out of position by the forwarded movement of the molar and bicusped teeth and so our greatest trouble with the conditions named is with the anterior teeth; finding them in many irregular positions, twisted, turned and over-lapped, caused by their being forced out of the position by the stronger teeth posterior to them.

These are the conditions we find starting and existing in the youth in this class of cases, conditions that should not be allowed to exist and which are sure to seriously handicap the individual; but they are often over-looked or are considered of minor importance, instead of being corrected at the outset and thus preventing the disease and abnormal conditions. If taken care of early this class of cases with the conditions of the gums can be corrected and will be found to return to a normal or nearly normal condition by the correction of mal-occlusion. Much depends upon the length of time the case has been allowed to remain in an uncorrected condition. It should be corrected as soon after the beginning of erruption of the permanent teeth as possible. This has more than one advantage. The case is not in its exaggerated form, the conditions of the gums are much better and in a growing condition, the process is not as solid and the teeth movement is much easier; the face grows into a normal condition, shape and expression instead of abnormal, and derives a degree of perfection, impossible if the case is delayed and corrected later when the tissues should have obtained their growth.

Not only that but the case is much harder to correct, much more irritating to the patient and exceedingly exhausting to the nervous system. The patient has been all this time growing into a more or less nervous condition. I don't wish to infer by this that a patient with mal-occlusion is always in a very nervous condition or that it is impossible to find a patient with mal-occlusion with fairly good nervous control, but I do make the statement that their condition would be much better if there was no mal-occlusion and is better after the case has been corrected. That cause of nervousness being eliminated.

When the patient is a mouth breather the lips being held apart by the position of the teeth, the patient will breathe through the mouth from force of habit, even though there is no obstruction to nose breathing. Besides in breathing through the mouth the fine particles of dust in the air are also very irritating. This condition is overcome with the correction of mal-occlusion and the mucous membrane will then return to its normal condition.

During the month of June, while writing this paper, I examined nearly one hundred school children in order to substantiate my statements, if it were possible. In age they were from five years to seventeen and grades from first to ninth.

There is some contention as to whether we find much mal-occlusion among the first or deciduous teeth. I am not prepared for that question, for the children examined were, without exception, too old. The youngest one was five years and this child had already cut its first permanent molars, and at that age the permanent teeth are exerting an influence in the matter of occlusion. But if there is mal-occlusion of the deciduous teeth, the above conditions will be found to be present in accordance to the degree of mal-occlusion.

It seems to me we could readily answer the question if enough children at the proper age (from three to five years old) were examined to give us an average. This is difficult for at that early age, they seldom enter our offices for treatment.

In my examinations I found the youngest children with the mouth in fairly good condition. There were a few exceptions. One little girl could only touch her first permanent molars in closing the mouth. Another a mouth breather, only her first permanent molars and centrals. In still another case, the lower right cuspid tooth was entirely exposed on the labial surface. The gums and process having receded until the end of the root was exposed, but the tooth had abscessed for a long time which must have a great deal to do with the condition. In quite a number of cases I found abscesses, showing that not enough attention is paid to filling and treating the deciduous teeth. Think of allowing those teeth to become badly decayed and nerves exposed subjecting the child to toothache and afterwards an abscess which is allowed many times to remain until the teeth are lost and the permanent ones erupt, at which time the condition generally is overcome; but, can we say without injury to the second teeth and tissues?

In the case examined at five and six years, I found about one third of them with very good articulation. From seven to nine there was not as good occlusion present, but I did not find as bad conditions present as regards decay, for the requisite number of permanent teeth were generally erupted, and there had been some attempt at filling, while in the younger children there was no sign of filling or treatment, and of course the teeth were not as good in quality to start with. The conditions of the gums I found growing worse as mal-occlusion advanced, more cases where the gums and process were receding. From nine to twelve the mal-occlusion in the cases present was worse and the gums correspondingly worse, especially in the case where the teeth were not kept clean. At this age the detrimental results of early extractions were apparent; the anterior teeth on the side where extracting had been done were not forced as far forward as they should be and the space left by extraction was closing up. In two cases the occlusion was shown to have been good but when the teeth were extracted the space was slowly being closed up, resulting in mal-occlusion of a pernicious kind.

Promiscuous extraction cannot be too greatly condemned; but there are conditions where it is necessary, indeed I believe patients would not demand extraction if the trouble arising from it was fully explained to them. But if it is found necessary to extract on account of the extent of decay there is no excuse for not retaining the space and allowing the child to grow in normal condition, getting the best results from developement of the jaw, and after a time the space may be bridged over when we may expect good results.

From the ages of twelve to seventeen and beyond, I find the mal-occlusion in a more advanced stage, and of necessity advancing the troubles. Also with mal-occlusion the habits are formed, which have a tendency to help on the existing condition.

At these periods we have the adenoid conditions generally gone but catarrhal conditions taking their place, if not also existing before, which helps to maintain the breathing habits formed. Also with mal-occlusion at these stages, we have deposits on the teeth which we have not had to contend with before, and which helps to cause our worse trouble, abscesses, pyorrhea, loss of tissue, loss of tooth.

The conclusion seems to indicate that these conditions and their relations are not receiving a proper amount of attention from the general practitioner. They are conditions which are the precursors of a great deal of trouble which we are at times sorely tried in explaining to our patients, and which, if patients understood correctly, they would demand treatment at a time when success can be assured. It is our duty, as practitioners, to diagnose and overcome these inimical tendencies before they produce results that are difficult if not impossible to cure.

### DISCUSSION.

Dr. O. W. White. I only wish to call attention of those who have had the privilege of looking at the patient which Dr. Rogers had in her clinic yesterday morning.

This was a case which illustrates this subject to perfection.

It was a bad case of mal-occlusion and was a severe case of pyorrhoea and a severe case of caries. The posterior teeth were all destroyed by decay, and I think there are very few who would realize the condition of the mouth when Dr. Rogers first took hold of it. It must have been in a frightful condition. We find this in all cases where we have mal-occlusion to any great extent, because I think in all the pyorrhoea cases without a doubt mal-occlusion is a primary cause. The teeth are not true to their function. They are just the same as any other organ of the body when it is thrown out of position, its normal circulation is destroyed which stops the nutriment going to it and the waste products from getting away, in such cases we have congested conditions causing decay and inflammation and we are bound to have an unhealthy organ. The largest percent of the mouths that are presented to our offices have malformation in a greater or less degree and they have diseased gums to a corresponding degree.

I am of the opinion that our only salvation is prevention of these diseases by removing the impending causes.

